# Linked survey and statutory health insurance data evaluating healthcare utilization patterns and associated factors of persons with diabetes in Germany – latent class analysis

**DOI:** 10.1038/s41598-025-95514-9

**Published:** 2025-04-04

**Authors:** Ute Linnenkamp, Inga Deininghaus, Veronika Gontscharuk, Silke Andrich, Manuela Brüne, Nadezda Chernyak, Johannes Kruse, Mickaël Hiligsmann, Barbara Hoffmann, Andrea Icks

**Affiliations:** 1 Institute for Health Services Research and Health Economics, Centre for Health and Society, Medical Faculty and University Hospital Düsseldorf, Heinrich Heine University Düssseldorf, Moorenstraße 5, 40225 Düsseldorf, Germany; 2 Institute for Health Services Research and Health Economics, German Diabetes Center, Leibniz Center for Diabetes Research at Heinrich Heine University Düsseldorf, Düsseldorf, Auf’m Hennekamp 65, 40225 Düsseldorf, Germany; 3 German Center for Diabetes Research (DZD), Partner Düsseldorf, Ingolstaedter Landstraße 1, 85764 München - Neuherberg, Germany; 4 Department of Health Services Research, CAPHRI Care and Public Health Research Institute, Maastricht University, Maastricht, The Netherlands; 5 Clinic for Psychosomatic Medicine and Psychotherapy, University Clinics Gießen and Marburg, Friedrichstraße 33, 35392 Giessen, Germany; 6 Institute for Occupational, Social and Environmental Medicine, Centre for Health and Society, Medical Faculty and University Hospital Düsseldorf, Heinrich Heine University Düsseldorf, Gurlittstr. 55/II, 40223 Düsseldorf, Germany

**Keywords:** Health care economics, Health services, Public health

## Abstract

Persons with diabetes mellitus have complex healthcare needs. Existing disease management programmes (DMPs) are based on a one-size-fits-all approach. However, individuals might require more individualised care. This study aims to identify groups with different patterns of healthcare utilization among people with diabetes in Germany and factors associated with these different patterns. A cross-sectional survey was conducted among a random sample from a statutory health insurance (SHI) with diabetes (n = 1332) and linked to longitudinal SHI data. Latent class analysis was used to identify subgroups with similar patterns of healthcare utilization and factors associated with different patterns. Four patterns of healthcare utilization were identified among people with diabetes: ‘low users’ (20.8% of the total sample); ‘low users with ophthalmologist visit’ (45.2%); ‘high users’ (26.5%); and ‘high users with mental health care’ (7.5%). The classes differed significantly in age, sex, type, duration and severity of diabetes, DMP membership, diabetes training, health-related quality of life, and prevalence of depression. The ‘high users with mental health care’ class was for example younger, more female, had a lower quality of life and the highest prevalence of depression. This study may provide a first basis for thinking about targeted care in Germany beyond DMPs.

**Supplementary Information:**

The online version contains supplementary material available at 10.1038/s41598-025-95514-9.

## Introduction

As diabetes mellitus prevalence increases worldwide^1^, accompanying comorbidities reduce persons’ life expectancy and health-related quality of life (HRQL). As a result, health expenditures due to diabetes are rising^2^. Furthermore, persons with diabetes utilize most healthcare services more often than the general population^3–6^.

Within Germany, provision of care takes place in a universal multi-payer healthcare system paid for by a combination of statutory health insurance (SHI) and private health insurance. Since 2009, health insurance is compulsory for the whole population in Germany. The majority of the population (around 85%) is insured with a SHI^7^. The reimbursement for outpatient care by the SHI is a uniform fee schedule and negotiated between SHIs and regional associations of physicians and is used to reimburse general practitioners and specialists under SHI. As part of the SHI contract, ambulatory physicians are required to join these regional associations, which act as financial intermediaries between physicians and statutory health insurance providers. The associations receive monies from statutory health insurance providers in the form of annual capitations. The physicians then bill the associations according to the SHI fee schedule. Physician payments are limited to predefined quarterly maximums for the number of patients and treatment points per patient that can be reimbursed. Physicians who exceed quarterly thresholds are paid considerably less for additional services. Once physicians reach the thresholds, they may postpone non-urgent patient visits, which may result in longer appointment wait times. Inpatient care is paid per admission through a system of diagnosis-related groups (DRGs), which are revised annually^7^. Disease management programmes (DMPs) have been implemented in the German healthcare system since 2002 to improve the quality and efficiency of chronic disease care^8^. In Germany, DMPs are offered to all patients and are centred around a certain disease, assuming one size fits all. However, it is discussed if patients would benefit from more individualized care approaches and if a shift should be made from a disease-centred approach to a more person-centred approach^9^. Identifying specific target-groups to optimize diabetes care might improve diabetes-related health outcomes, reduce comorbidities, and in the long run reduce associated costs. It is therefore crucial to understand both differences in patterns of healthcare utilization and factors that contribute to these differences. Identifying possible target groups is the first step in developing target group specific interventions. One possibility of identifying these target groups is population segmentation^10^: Segmentation is grouping people based on, for example, how often they use certain health services. This might for example help to identify those who frequently utilize services and to provide them with proactive care to effectively manage their conditions and avoid for example unnecessary hospital admissions. On the other hand, this gives also the opportunity to identify individuals who utilize healthcare services infrequently and engage them with preventive strategies if necessary to diagnose issues at an early stage. By focusing on the optimal level of intervention for each group, healthcare systems might enhance outcomes, reduce costs, and create a more efficient and patient-centered approach to the management of chronic diseases. A possible approach for grouping a population is latent class analysis (LCA). LCA identifies the presence of qualitative different subgroups (i.e., latent classes) within a heterogeneous population and can be seen as a parametrical alternative to cluster methods for multivariate categorical data. Individuals within the same class are similar with respect to observed variables while individuals in different classes differ considerably. LCA results in estimates of two types of parameters: (i) latent class membership probabilities and (ii) item-response probabilities on each observed variable in each latent class. Moreover, LCA estimates individual posterior probabilities to belong to each latent class based on the observed variables. LCA with covariates extends the LCA model by relating the probability of class membership to a set of predictors. For more information see, e.g. Collins and Lanza (2009)^11^.

So far, only few studies have used LCA to analyse healthcare utilization profiles. Two Dutch studies found distinct different profiles of utilization among persons with diabetes. Profiles were for example associated with persons’ age or number of comorbidities^12,13^. Similarly, an analysis in Singapore has investigated patterns of healthcare utilization, diabetes-related complications, and 4-year all-cause mortality from 2013 to 2016 among 71,125 patients with type 2 diabetes mellitus^14^. They found five distinct classes of healthcare use with two classes who might have a need to be screened for comorbid psychiatric disorders such as depression.

These differences in utilization patterns are related to a number of associated factors, both of a clinical and of a socio-demographic nature^12–14^. Healthcare utilization can be well derived from statutory health insurance (SHI) data. Using SHI data to assess healthcare utilization especially for longer observation periods (> 3 month) seems to be more reliable than self-reported data^15,16^. However, most socio-demographic variables are not included in SHI data (e.g. duration of education). To have a more holistic approach when analysing associated factors, it is thus necessary to individually link data sources that include information on both clinical but also sociodemographic variables. The aims of this study were to identify (i) patterns of healthcare utilization and (ii) factors associated with these different patterns of healthcare utilization in a group of persons with diabetes from a German SHI by linking survey data with SHI data on an individual level.

## Methods

### Study design and population

Within the DiaDec Study: “Quality of life, adverse outcomes, healthcare utilization, and costs in patients with diabetes: the role of depression” we conducted a cross-sectional postal survey in 2013 among randomly selected individuals with diabetes insured with the pronova Betriebskrankenkassen (BKK) and later linked the data on an individual level to longitudinal SHI data of the persons who provided written informed consent for the linkage. In 2013, 636.451 individuals were insured by pronova BKK which is a nationwide SHI. Compared to other SHIs the pronova BKK insures rather younger persons^17^. SHI data was available for 9 quarters in total (four quarters before the survey quarter, the survey quarter and four quarters following the survey quarter). The SHI identified all insured persons with diabetes in February 2013, using data from the year 2011, the most recent year with complete information available. Diabetes was identified either as: (i) a regular documentation of a diabetes diagnosis (ICD-10 E10-E14) in three of four quarters; (ii) at least two prescriptions of antihyperglycemic medication (Anatomical Therapeutic Chemical (ATC) code A10) within 2011; or (iii) a single A10 prescription combined with a diabetes diagnosis or a single A10 – prescription combined with either blood glucose or HbA1c measurement in the same quarter^18,19^.

In total 1,860 persons sent back their questionnaire and gave informed consent to use their SHI data (response rate: 51%). Responders and non-responders differed in age and sex, with older persons being more likely to respond than younger persons and females being less likely to respond than males. However, we found no difference between responders and non-responders with respect to a history of depression diagnosis^20^. We excluded (i) responders who had an insurance period of less than 1065 days (which corresponds to the entire observation period including tolerable insurance gaps) (n = 201), e.g., because the person switched health insurance during that time, and (ii) persons who did not know their type of diabetes or did not provide information on their type of diabetes (n = 118). We (iii) excluded persons who provided incomplete information in the questionnaire for any of the associated factors (n = 209). Ethical approval was obtained from the ethics committee of the Heinrich Heine University Düsseldorf and the research has been performed in accordance with the Declaration of Helsinki.

### Measurement of healthcare utilization

The variables of healthcare utilization referred to the period of four quarters after the survey and were taken from SHI data. The SHI data comprised details of outpatient treatment, including recorded diagnoses and services accounted for, as well as data on inpatient care, including both diagnoses recorded and services charged, and length of hospital stay. Additionally, the dataset contained information on prescriptions dispensed at pharmacies, as well as information on therapeutic appliances and medical aids. We assessed general practitioner (GP) visits, visits at specialized outpatient services (cardiology, neurology, nephrology, ophthalmology, specialists dealing with mental illnesses), emergency care and hospital admissions. As in LCA measurable variables should be categorical with two or more categories per variable^21^ we categorized the healthcare utilization variables as follows:

The number of annual GP contacts for patients with diabetes in the literature is varying largely: E.g. in the CODE-2 study, the average number of contacts ranged from 20 to 34^22^, while the KoDim study found that patients over the age of 60 had an average of 12 contacts^19,23^. Data from the AOK and the Barmer for the general population yielded an average of seven to ten GP consultations per year, respectively^24,25^. We thus decided to take a data-based approach: Within our sample we found a mean number of 13 visits to the GP and a median of 15 visits and thus decided for the cut-off of 14 visits. We thus classified the number of GP visits in frequent (> 14 visits/year) and infrequent (≤ 14 visits/year).

Utilization of emergency care services, specialists’ consultations (i.e., cardiology, mental health) and hospital admissions were dichotomized as yes/no with ≥ 1 visits/year referring to yes. Visits to the neurologist and nephrologist were rather low in our sample even though both specializations are relevant to patients with diabetes. We combined visits to these specializations into one variable, which was then dichotomized as yes/no. In accordance with the DMP recommendations, patients with an elevated risk profile are required to visit an ophthalmologist at regular intervals, with the frequency depending on the specific risk profile. This may range from a visit every two years to a visit annually, or even more frequently, depending on the individual circumstances. Thus, we categorized visits to the ophthalmologist as: 0 visits/year, 1 visit/year, ≥ 2 visits/year.

### Measurement of associated factors

Based on the literature, we selected factors which might be associated with healthcare utilization among patients with diabetes and defined the following three thematic groups of variables:

i. *Sociodemographic factors*^19,23,26–32^ Socio-demographic factors as age, sex, employment status (yes/no), having a partner (yes/no), country of birth (Germany/other) and education were assessed in the survey. Education was measured using the International Standard Classification of Education (ISCED) and respondents were categorized according to years of education (10 years or less, 11–13 years, 14 years or more).
ii. *Diabetes-related factors*^33–35^ We assessed the type of diabetes (type 1, type 2) and duration since diagnosis of diabetes (< 10 years, ≥ 10 years) within the survey. Involvement in a DMP for diabetes, participation in diabetes training and the adapted Diabetes Complications Severity Index (aDCSI) were retrieved from SHI data during the survey quarter and the four quarters before. The aDCSI is based on the following diabetes-associated comorbidities^36^ : Retinopathy, Nephropathy, Neuropathy, Cerebrovascular diseases, cardiovascular diseases, Peripheral arterial occlusive disease and metabolic complications. The comorbidities are identified using ICD-10 codes which have been recorded in the SHI data. The severity of the comorbid diseases is rated with one or two points based on the severity. An exception to this is diabetic polyneuropathy, which can only be rated with one point. Thus, in total the aDCSI can have a value between 0 and 13.
iii. *Health related Quality of life (HRQL) and depression*^28,37^ HRQL was examined by the Short Form-12 Health Survey (SF-12), a multipurpose generic measure of health status^38^. The SF-12 has a Physical Component Summary (PCS) and a Mental Component Summary (MCS). Each can have values between 0 and 100. The higher the value, the better the reported quality of life. Depression was assessed by retrieving diagnoses from the SHI data. An ICD-10 code for the diagnosis of unipolar depression (F32.0–F32.9 (Depressive episode), F33.0–F33.9 (Recurrent depressive disorder), F34.1 (Dysthymia), F38.1 (Other recurrent mood [affective] disorders) and F41.2 (Mixed anxiety and depressive disorder)) during the survey quarter and/or during the four quarters before the survey quarter was required in either inpatient or outpatient data. The diagnosis needed to be coded as confirmed (ICD-10 code with suffix g-gesichert for confirmed diagnoses) to be considered.

### Statistical analysis

Descriptive statistics are provided as frequencies and percentages or mean values and standard deviations depending on the nature of variables. All quantitative analyses were performed with SAS software, V.9.4 (SAS Institute Inc., Cary, NC, USA) using the package PROC LCA for the LCA analysis.

As a person-centered approach, LCA divides populations into classes, with individuals within a class being relatively homogeneous, yet distinct from those within other classes^39^. According to Nylund^40^, our sample size is large enough (i.e., N > 1000) to adequately fit an LCA model. The first step is to determine the optimal number of classes. To select a suitable model, we included the study population with complete SHI data irrespective of missing values in the covariates assessed in the survey (n = 1659) as it is crucial to include as many people as possible to determine the optimal number of classes^41^. Bayesian information criterion (BIC) and adjusted Bayesian information criterion (aBIC) indicate a better fit when their values are lower. High separation of classes is indicated by a (relative) entropy close to one. In order to assess the overall model fit, we used a likelihood ratio chi-square test, with a significant test indicating poor data adaptation. The choice of the final number of classes was based on model fit indicators in compliance with a non-significant (at level 5%) likelihood ratio chi-square test as well as interpretability of the latent classes. Afterwards the LCA with the final number of latent classes was performed with all covariates simultaneously to describe classes and to analyze the factors associated with the identified classes.

## Results

### Description of the study population

Table 1 describes the 1332 participants included in the final analysis (i.e., LCA with covariates) and their characteristics. The mean age was 67 years and the majority were men (63%). Half of the population had diabetes for less than 10 years, and the majority had type 2 diabetes mellitus (91%) and was enrolled in a DMP (84%). Almost every sixth person had a diagnosis of depression. Almost one third of our study population was hospitalized at least once during the observation period. One in seven took up services from the mental health sector at least once a year. Only one in five persons visited the cardiologist during the observation period and only one in nine the Nephro- or Neurologist. Around one third of the study population did not visit the ophthalmologist, one third went once during the observation period, and one third went more than once.

**Table 1:** Participants’ characteristics and characteristics of classes.

	Study sample
Characteristics n (%)/(M ± SD)	
Sample size (%)	1332 (100)
Age (years)	66.7 ± 10.1
Sex (female)	491 (36.9)
Years of education	
≥ 14 years	298 (22.4)
11–13 years	767 (57.6)
≤ 10 years	267 (20.0)
Country of birth (Germany)	1,185 (89.0)
Partner (yes)	1,068 (80.2)
Employment (yes)	358 (26.9)
Type of diabetes (type 2)	1,211 (90.9)
Diabetes duration (≥ 10 years)	670 (50.3)
aDCSI	2.5 ± 2.0
DMP Member (yes)	1,121 (84.2)
Diabetes training (yes)	189 (14.2)
PCS12	41.8 ± 10.9
MCS12	50.3 ± 10.5
Depression (yes)	234 (17.6)

GP visits (> 14)	573 (43.0)
Emergency care (≥ 1)	173 (13.0)
Hospitalization (≥ 1)	393 (29.5)
Cardiologist visits (≥ 1)	298 (22.4)
Nephro-/Neurologist visit (≥ 1)	147 (11.0)
Ophthalmologist visits	
0	395 (29.7)
1	490 (36.8)
≥ 2	447 (33.6)
Mental health sector visits (≥ 1)	181(13.6)

M, mean; SD, standard deviations; aDCSI, adjusted diabetes severity index; DMP, disease management program; GP, general practitioner; PCS12, physical component summary score of the short form-12 health survey; MCS12, mental component summary score of the short form-12 health survey.

### Groups with different patterns of healthcare utilization

**Fig. 1:**
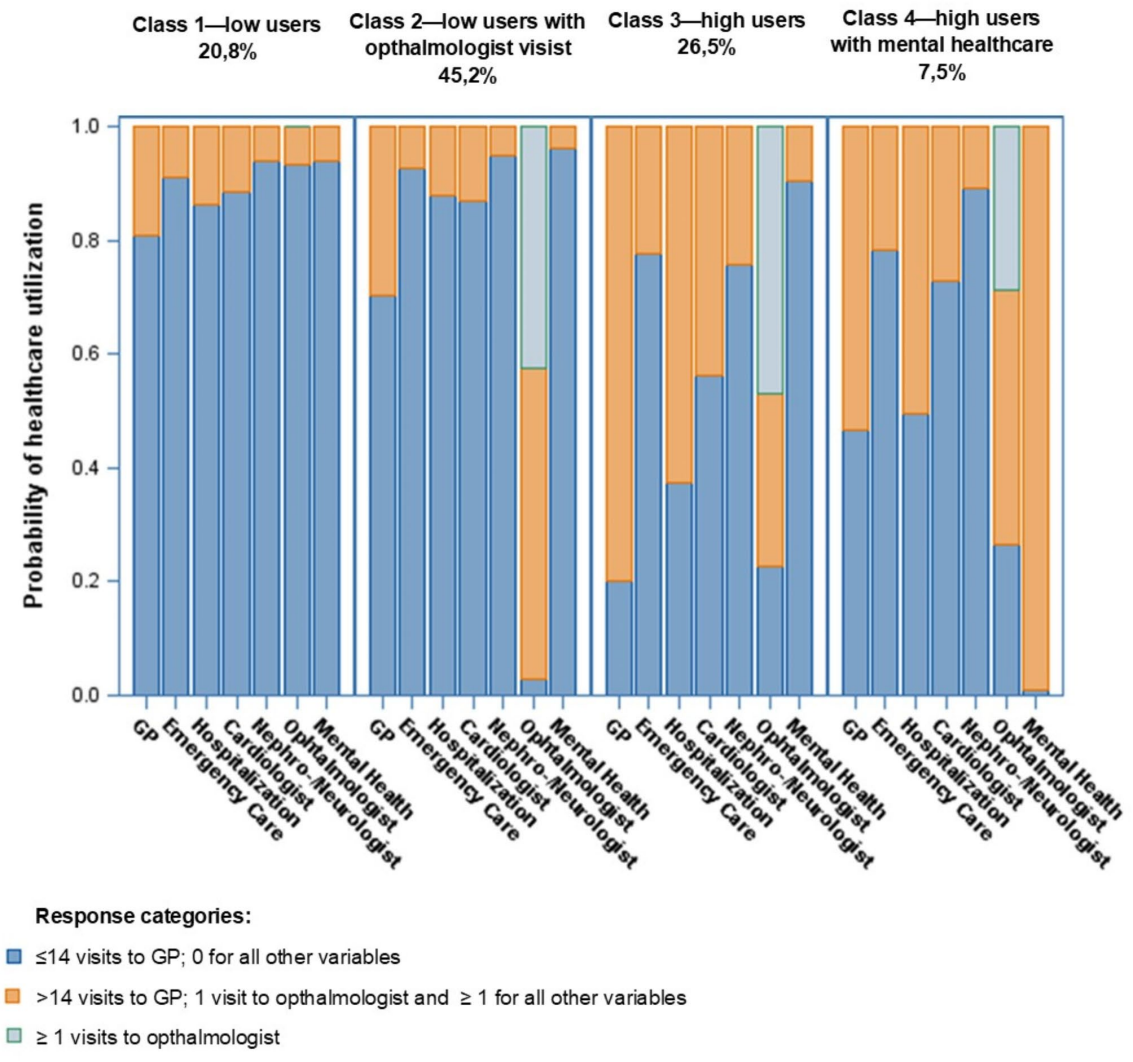
Probability of healthcare utilization among the different classes.

The model with two classes had the lowest information criteria (AIC = Akaike information criterion BIC = Bayesian information criterion, CAIC = Consistent Akaike information criterion, aBIC = Sample size adjusted BIC). Differences between models were small (refer to Online Appendix Table 1). Only in the model with four classes was the likelihood ratio chi-square test (as a goodness-of-fit test) not significant at the 5% level, indicating that the models with one to three classes do not represent our data well. Entropy was largest in the model with four classes. Furthermore, we compared the different LCA models including covariates and the model with four classes was the most meaningful model. The identified classes represent four different profiles of healthcare utilization among people with diabetes as shown in Fig. 1 and Online Appendix Table 3:

1. *Low probability of healthcare utilization (low users):* Persons in class one had in general a low probability of healthcare utilization, with infrequent visits to healthcare providers other than their GP. We estimated that only 6%-14% sought health services beyond contacting their GP. Further we estimated, that over 80% of this group had ≤ 14 consultations with their GP during the observation period (refer to Online Appendix Table 3).
2. *Low probability of healthcare utilization but high probability to visit the ophthalmologist (low users with ophthalmologist visits):* Persons in class two had a low probability of utilising healthcare in general, similar to persons in class one. We estimated i.e. only 5–13% of persons in that class utilized other health services than their GP or ophthalmologist. However, we found that almost all persons in that group consulted an ophthalmologist at least once (97%), with around 40% consulting the ophthalmologist two or more times. Additionally, we found that 70% of persons in class two had ≤ 14 consultations with their GP during the observation period.
3. *Highest probability of healthcare utilization except for visits to the ophthalmologist or mental health sector (high users):* The probability of using health services was high among persons in class three. They had the highest probability of visiting a cardiologist (44%) or nephrologist/neurologist (24%), using emergency care (22%) and being admitted to hospital (63%) during the observation period. Moreover, we estimated, that 80% of persons in this group consulted their GP more than 14 times.
4. *High probability of healthcare utilization and highest probability of utilizations from the mental health sector (high users with mental health care):* Persons in class four had a high probability of healthcare utilization, comparable to persons in class three. Additionally, they demonstrated the highest probability seeking treatment form mental health specialist (estimates almost 100%). The probability of emergency care utilization was around 20% in this group and hospitalization probability during the observation periods exceeded 50%. Yet, only 53% of this group consulted their GP more than 14 times.

### Factors associated with the different utilization patterns

The characteristics of participants assigned to the different classes can be found in Online Appendix Table 2. We found that age, sex, type of diabetes, diabetes duration, aDCSI, DMP membership, diabetes training, PCS12, MCS12 and depression differ between at least two of the four classes of healthcare utilization (Tables 2 and 3). The covariates ‘years of education’ ‘country of birth’, ‘partner’, and ‘employment’ showed no significant associations.

**Table 2:** Results of the LCA with covariates (n = 1332).

Comparison group	Low users with ophthalmologist visits class 2		High users class 3		High users with mental healthcare class 4		High users class 3		High users with mental healthcare class 4		High users with mental healthcare class 4		Type III test
Reference group	Low users-class 1						Low users with ophthalmologist visits class 2				High users class 3		
	OR	CI 95%	OR	CI 95%	OR	CI 95%	OR	CI 95%	OR	CI 95%	OR	CI 95%	p-value
Age (years)	**1.03**	**[1.01; 1.05]**	**1.02**	**[1.00; 1.04]**	1.00	[0.97; 1.02]	0.99	[0.98; 1.01]	**0.97**	**[0.94; 0.99]**	**0.97**	**[0.95; 1.00]**	**0.0022**
Sex (female vs. male)	1.26	[0.98; 1.63]	1.10	[0.80; 1.50]	**1.70**	**[1.10; 2.61]**	0.87	[0.67; 1.12]	1.34	[0.90; 2.01]	**1.55**	**[1.01; 2.36]**	0.1721
Years of education (≤ 10 vs. ≥ 14 years)	1.14	[0.77;1.69]	1.32	[0.82;2.10]	1.39	[0.70;2.77]	1.15	[0.78; 1.70]	1.22	[0.64;2.33]	1.05	[0.53;2.08]	0.8076
Years of education (11–13 years vs. ≥ 14 years)	1.15	[0.87;1.52]	1.29	[0.90;1.84]	1.60	[0.93;2.78]	1.12	[0.82; 1.53]	1.39	[0.82;2.37]	1.25	[0.71;2.17]	0.4052
Country of birth (Germany vs. not Germany)	0.90	[0.61; 1.31]	0.97	[0.61; 1.53]	0.86	[0.47; 1.58]	1.08	[0.73; 1.58]	0.96	[0.54; 1.68]	0.89	[0.49; 1.61]	0.9800
Partner (yes vs. no)	1.07	[0.80; 1.44]	1.30	[0.91; 1.87]	1.51	[0.91; 2.52]	1.21	[0.90; 1.64]	1.41	[0.87; 2.27]	1.16	[0.70; 1.91]	0.5020
Employment (yes vs. no)	0.86	[0.64; 1.15]	0.70	[0.48; 1.01]	0.82	[0.49; 1.37]	0.81	[0.58; 1.13]	0.95	[0.58; 1.55]	1.17	[0.69; 1.98]	0.6742
Type of diabetes (Type 2 vs. Type 1)	1.14	[0.75; 1.74]	**1.82**	**[1.02; 3.23]**	0.66	[0.34; 1.30]	1.59	[0.96; 2.65]	0.58	[0.31; 1.09]	**0.37**	**[0.18; 0.74]**	0.1756
Diabetes duration ((≥ 10 years vs. < 10 years)	**1.56**	**[1.22; 1.98]**	**1.49**	**[1.12; 2.00]**	0.95	[0.62; 1.45]	0.96	[0.76; 1.22]	**0.61**	**[0.41; 0.90]**	**0.63**	**[0.42; 0.96]**	**0.0123**
*aDCSI*	**1.17**	**[1.09; 1.27]**	**1.57**	**[1.44; 1.70]**	**1.37**	**[1.21; 1.55]**	**1.34**	**[1.26; 1.42]**	**1.17**	**[1.04; 1.30]**	**0.87**	**[0.78; 0.98]**	** < 0.0001**
DMP member (yes vs. no)	**1.94**	**[1.45; 2.58]**	**2.09**	**[1.44; 3.05]**	1.52	[0.89; 2.58]	1.08	[0.76; 1.53]	0.79	[0.47; 1.32]	0.73	[0.42; 1.27]	**0.0041**
Diabetes Training (yes vs. no)	1.24	[0.85; 1.81]	**1.65**	**[1.07; 2.54]**	1.21	[0.69; 2.13]	1.33	[0.96; 1.85]	0.98	[0.59; 1.62]	0.74	[0.44; 1.23]	0.2083
PCS12	1.00	[0.99; 1.02]	**0.97**	**[0.96; 0.99]**	**0.98**	**[0.96; 1.00]**	**0.97**	**[0.96; 0.98]**	**0.97**	**[0.95; 0.99]**	1.00	[0.98; 1.03]	**0.0001**
MCS12	1.01	[0.99; 1.02]	1.00	[0.98; 1.02]	**0.96**	**[0.94; 0.97]**	0.99	[0.98; 1.01]	**0.95**	**[0.93; 0.97]**	**0.96**	**[0.94; 0.97]**	**0.0002**
Depression (yes vs. no)	0.98	[0.67; 1.43]	1.51	[0.99; 2.30]	**6.29**	**[3.85; 10.29]**	**1.54**	**[1.11; 2.14]**	**6.40**	**[4.18; 9.79]**	**4.17**	**[2.68; 6.48]**	** < 0.0001**

Significant values are in bold.

OR, odds ratio (in age, aDCSI, PCS12 and MCS12 corresponding to one unit change); CI 95%, 95% confidence intervals; aDCSI, adjusted diabetes severity index; DMP, disease management program; PCS12, physical component summary score of the short form-12 health survey; MCS12, mental component summary score of the short form-12 health survey, significant results (*p* < 0.05).

**Table 3:** Description of class characteristics compared to the other classes.

Selected characteristics	Typical person in….			
	Class 1-Low users	Class 2-low users with ophthalmologist visits	Class 3-high users	Class 4-high users with mental healthcare
Age (years)	*Younger*	*Older*	*Older*	*Younger*
Sex (female)	Male	Female	Male	*Female*
Type of diabetes	*Less than 90% T2DM*	*More than 90% T2DM*	*More than 90% T2DM*	*Less than 90% T2DM*
Diabetes duration	*Shorter duration*	*Longer duration*	*Longer duration*	*Shoter duration*
aDCSI^*#*^	*Low*	*Low*	*High*	*High*
DMP Member	*Less*	*More*	*More*	*Less*
Diabetes training	Less	More	More	Less
PCS12*	*High*	*High*	*Low*	*Low*
MCS12*	*High*	*High*	*Low*	*Low*
Depression	*No*	*No*	*Yes*	*Yes*

Significant results (Table 2) are marked in italics; the results were extracted from the LCA main analysis (Table 2 and Online Appendix Table 2).

^#^low aDCSI referring to a low severity of diabetes complications.

*high PCS12 or MCS12 referring to high self-reported quality of life.

Class one, consisted of rather younger study participants who were significantly less likely to be members of a DMP compared to class two and three. Fewer persons in class one attended diabetes training compared to class three (OR 0.61, 95%-CI [0.39; 0.93]). Additionally, persons diagnosed with diabetes for less than 10 years were significantly more likely to be in class one compared to class two and three. The chance of study participants with lower aDCSI to belong to class one was significantly higher than for other classes.

Class two included persons who had a significantly longer duration of diabetes compared to persons from the classes one and four (OR 1.56, 95-CI [1.22; 1.98], and 1.64, 95%-CI [1.11; 2.43], respectively). In addition, class two included persons who had a higher probability of being enrolled in a DMP compared to those in class one (OR 1.94, 95%-CI [1.45; 2.58]). Additionally, class two included more persons with lower rates of depression and a better quality of life than those in class four. Moreover, class two included persons with a significantly lower aDCSI than classes three and four.

Compared to the other classes, class three was comprised of older persons who had been diagnosed with diabetes for a longer period of time than those in classes one and four. Additionally, the chance to belong to class three was significantly higher for people with a high aDCSI compared to the other classes, and they also reported a lower quality of life according to the PCS12 than class one and class two. Class three included a significantly higher proportion of persons diagnosed with depression than class two.

Class four had the highest proportion of females. Class four less persons with a diabetes duration of more than 10 years and a lower age compared to persons in classes two and three. Nevertheless, class four included persons with a significantly higher aDCSI compared to class one or two (OR 1.37, 95%-CI [1.21; 1.55] and 1.17, 95%-CI [1.04; 1.30]). Class four included persons with a significantly lower quality of life than class one and two, moreover class four had a significantly higher proportion of persons diagnosed with depression than the other classes (OR 6.29, 95%-CI [3.85; 10.29]; 6.40, 95%-CI [4.18; 9.79]; 4.17, 95%-CI [2.68; 6.48]).

## Discussion

To our knowledge, this study is one of the few that evaluates patterns of healthcare utilization among persons with diabetes and the first one carried out on a German sample. We identified four classes of healthcare utilization among persons with diabetes: two classes with low utilization patterns (‘low users’ and ‘low users with ophthalmologist visits’) and two classes with high utilization patterns (‘high users’ and ‘high users with mental health care’).

There were significant differences between the classes in their utilization of inpatient stays, emergency care, and visits to the cardiologist, ophthalmologist and the mental health sector. However, regarding associated factors, we could not find well defined groups of ‘typical person’.

Looking at utilization patterns, class one had a similar utilization pattern as class two except those participants in class two consulted an ophthalmologist at least once in the observation period. There were many differences concerning associated factors between the two classes. Persons in class one were generally younger, had a shorter duration of diabetes, a lower aDCSI, and were less likely to be a member of a DMP when compared to class two. Notably, in spite of different associated factors, including well-known risk factors such as age and duration of disease, the utilization patterns of class one and two were very similar. These results demonstrate that healthcare utilization can remain consistent even after many years of diabetes, resembling that of a person with a shorter duration of diabetes. A potential explanation might be that persons in class two were more adherent to their diabetes treatment (as demonstrated, for example, by participation in the DMP and regular visits to the ophthalmologist) and had despite a longer duration of diabetes less complications. The participation in a DMP could encourage regular checks of the eyes among that class.

When comparing class two and three we saw significant differences in the utilization behaviour. Class three had the highest utilization among study participants, except for visits to the ophthalmologist or mental health sector. When looking at associated factors however, members from class two and members from class three are most similar. They only differ significantly in three associated factors: depression, aDCSI and PCS12. There are no significant differences in socioeconomic factors, diabetes-related factors (despite aDCSI), probability of DMP membership, or participation in diabetes training. Although age and duration of diabetes do not differ significantly between classes two and three, their management of the disease appears to differ to some extent as reflected by the different utilization patterns., Significant differences in their diabetes complications (aDCSI) which might also lead to a better HRQL (PCS12) in class two, may explain the observed differences in the management of the disease. Noteworthy, the prevalence of depression is more than 50% higher in class three compared to class two. This might further explain the different patterns of utilization observed.

Classes three and four, the two classes with high utilization patterns had a very similar utilization pattern except that among class four 99% used services from the mental health sector and in class three only about 10%. Both classes had the highest prevalence of depression among the classes (20.8% and 73.3% respectively). This indicates that the presence of a diagnosed depression is also associated with an increased utilization of health services. This is in line with findings from several studies that found, that persons with diabetes and depression have higher healthcare utilization, including outpatient visits and hospital admission^42–44^. One possible explanation for these findings might be, that depression is associated with poor health behaviour and more comorbidities. Successful diabetes therapy may require attention to mental health. It may be worth considering to include the relevant services in the DMP. At the same time, it is important to note, that 60% of persons with a diagnosed depression are not in class four, even though this class had the highest prevalence of depression. One possible explanation could be, that class four represents the utilization behaviour of a group of persons with diagnosed depression that have a more severe form.

It is noteworthy that the covariates ‘years of education’, ‘country of birth’, ‘partner’, and ‘employment’ (all representing to some extend socioeconomic status (SES) of a person) showed no significant associations to class membership as it seems reasonable to assume that SES is likely to influence patterns of healthcare utilization. However, a paper by Baumert et al. (2021) addressing self-rated quality of care among persons with diabetes also found no significant differences in the self-rated quality of care between educational levels^45^. Similarly, findings of a study carried out with data from the AOK Niedersachsen, indicate that there are no statistically significant differences in the prevalence or progression over time of T2D comorbidities between different SES groups in Germany. No influence of SES on these aspects was observed in either cross-sectional or longitudinal analyses^46^.

Few previous studies have used a similar statistical method to evaluate healthcare utilization patterns of persons with diabetes^12–14^. Seng et al. (2020) classified persons with type 2 diabetes mellitus in Singapore according to socioeconomic and clinical characteristics using LCA and then compared healthcare utilization and the incidence of diabetes-related complications among these classes^14^. Similar to our results they identified two classes with a high prevalence of depression of which one consisted of young females. They also identified one class with a low overall healthcare use who were younger and had a shorter duration of diabetes which is in line with class one “low users” we identified.

El Fakiri et al. (2003) evaluated patterns of healthcare utilization among Dutch patients with diabetes^13^. They found two classes with high utilization patterns and two classes with low utilization patterns, which is consistent with our results. However, El Fakiri et al. only included services that were consulted by 10–90% of patients in their analysis to assess healthcare utilization. According to El Fakiri et al. comorbidities are related to class membership with high healthcare utilization. Our sample had the highest aDCSI score in the high user class which supports the Dutch findings. Similarly, El Fakiri et al. found an association between poor health perception and high healthcare utilization. In both the “high users” and "high users with mental healthcare", HRQL was lowest for our sample.

Van Dijk et al.^12^ conducted two LCAs to compare diabetes-related primary healthcare use with total primary healthcare use among patients registered in Dutch GP practices. They found that low diabetes-associated GP utilization correlated with low overall GP utilization, while high overall utilization did not necessarily correspond with high diabetes-associated GP utilization. Van Dijk et al. suggested that the Dutch DMP for diabetes, which focuses on diabetes specific care, might be limited in meeting the diverse needs of patients with multiple morbidities, which are common among persons with diabetes. Our findings indicate that primary care utilization alone may not be an adequate representation of healthcare utilization for persons with diabetes, as the largest disparities were found in inpatient visits, emergency care, mental health utilization, and visits to the ophthalmologist and cardiologist.

### Strengths and limitations

A major strength of our study was the individual linkage of survey data with SHI data. This allows a more detailed description of the identified classes with different healthcare patterns which would not be possible using SHI data only. The data set used for analysis is rather large, allowing for robust estimates including a total of nine quarters. The identification of associated factors is based on the first five quarters, variables of healthcare utilization are based on the following four quarters. Thus, one strength of the study was the longitudinal study design. Moreover, the response rate was reasonably high for a survey-based study (51.0%)^20^.

Furthermore, we were able to include a variety of different health services including but not limited to hospital admissions, emergency care and the mental health sector and are thus able to provide a wider insight into healthcare utilization patterns. Other studies have mainly focused on one sector of the health system (e.g. outpatient visits or primary care).

One limitation of our study is, that the SHI data used to generate some of the study variables originate from a single SHI provider. As a result, socio-demographic parameters of the study population, for example, may not be transferable to the overall population of Germany. Hoffmann and Icks (2012) could show that persons insured with any of the Betriebskrankenkassen (BKK) in the Bertelsmann survey were for example younger compared to persons insured with other health insurance providers and more women were insured with any BKK compared to other insurance providers^17^. The authors could further show, that the prevalence of diabetes varies among the different SHIs in Germany with persons insured with any of the BKK SHIs having a lower prevalence of diabetes compared to the whole population studied^17^. Additionally, we only considered individual factors that might influence healthcare utilization but not factors that might be related to the healthcare system. We could for example not assess whether the patient had to wait very long for an appointment with an ophthalmologist, if there were no appointments available within a decent distance or if the GP did not refer the patient to the ophthalmologist. Further, the definition of the aDSCI is based on diagnosis codes registered within the SHI for documentation and accounting purposes. We can thus not fully rule out a false classification of a disease. Another limitation is, that we could not differentiate which type of diabetes a person had based on SHI data, as we assume a relevant proportion of miscoding within the data with respect to the specific type of diabetes^47^. Lastly, we can only assess actual utilization but this does not indicate whether it is appropriate or not. There might be low users with well-managed diabetes who do not utilize healthcare frequently, as well as low users who are not concerned about their disease and thus have a low utilization pattern. High users may be people who adhere to all recommendations and are therefore well adjusted, or they may have complications due to inadequate adjustment and thus have a high utilization.

## Conclusion

Four distinct classes of persons with diabetes were identified based on their healthcare utilization patterns. The classes differed significantly in their utilization of inpatient stays, emergency care, and visits to the ophthalmologist, cardiologists and mental health sector. Based on classes three and four, we show that the existence of a diagnosis of depression and poor quality of life is associated with high utilization of health services, regardless of classical risk factors such as age or duration of illness. However, persons who utilize less health services are not necessarily healthier and might cause higher costs in a later stage of their disease. We have shown that the healthcare utilization within our sample of persons is rather heterogenous with two groups of high users. Understanding the diverse factors and unique profiles within this group is essential for tailoring effective healthcare strategies and optimizing resource allocation to address the complex needs of high healthcare users. These findings may provide a first basis for considering more targeted care beyond DMPs and for paving the way for a less rigid approach in the management of chronic diseases. The present results suggest that accounting for comorbid depression could be a step along the way. One approach could be considering to include the relevant services in the DMPs for diabetes.

## Electronic supplementary material

Below is the link to the electronic supplementary material.

Supplementary Material 1

## Data Availability

The data that support the findings of this study is not publicly available. Data can be shown by the corresponding authors at the Institute upon reasonable request.

## References

[1] Saeedi, P. et al. Global and regional diabetes prevalence estimates for 2019 and projections for 2030 and 2045: Results from the International Diabetes Federation Diabetes Atlas, 9th edition. *Diabetes Res. Clin. Pract.* **157**, 107843 (2019). 31518657 10.1016/j.diabres.2019.107843

[2] Williams, R. et al. Global and regional estimates and projections of diabetes-related health expenditure: Results from the International Diabetes Federation Diabetes Atlas, 9th edition. *Diabetes Res. Clin. Pract.* **162**, 108072 (2020). 32061820 10.1016/j.diabres.2020.108072

[3] Müller, N. et al. Healthcare utilization of people with type 2 diabetes in Germany: an analysis based on health insurance data. *Diabet. Med. J. Br. Diabet. Assoc.* **32**(7), 951–957 (2015). 10.1111/dme.12747 25781644

[4] Jacobs, E. et al. Healthcare costs of Type 2 diabetes in Germany. *Diabet. Med. J. Br. Diabet. Assoc.* **34**(6), 855–861 (2017). 10.1111/dme.13336 28199029

[5] Ulrich, S. et al. Cost burden of type 2 diabetes in Germany: Results from the population-based KORA studies. *BMJ Open* **6**(11), e012527 (2016). 27872118 10.1136/bmjopen-2016-012527 PMC5129071

[6] Hauner, H., Köster, I. & von Ferber, L. Outpatient care of patients with diabetes mellitus in 2001. Analysis of a health insurance sample of the AOK in Hesse/KV in Hesse. *Dtsch Med. Wochenschr.* **128**(50), 2638–2643 (1946). 10.1055/s-2003-45484 14673739

[7] Busse, R., Blümel, M., Knieps, F. & Bärnighausen, T. Statutory health insurance in Germany: A health system shaped by 135 years of solidarity, self-governance, and competition. *Lancet* **390**(10097), 882–897 (2017). 28684025 10.1016/S0140-6736(17)31280-1

[8] Busse, R. Disease management programs in Germany’s statutory health insurance system. *Health Aff. (Millwood).* **23**(3), 56–67 (2004). 15160803 10.1377/hlthaff.23.3.56

[9] Geurten, R. J. et al. Identifying and delineating the type 2 diabetes population in the Netherlands using an all-payer claims database: Characteristics, healthcare utilisation and expenditures. *BMJ Open* **11**(12), e049487 (2021). 34876422 10.1136/bmjopen-2021-049487 PMC8655569

[10] Chong, J. L., Lim, K. K. & Matchar, D. B. Population segmentation based on healthcare needs: A systematic review. *Syst. Rev.* **8**(1), 202 (2019). 31409423 10.1186/s13643-019-1105-6 PMC6693177

[11] Collins, L. M. & Lanza, S. T. Latent Class and Latent Transition Analysis: With Applications in the Social, Behavioral, and Health Sciences [Internet]. 1st ed (Wiley, 2009) [cited 2024 Oct 11]. (Wiley Series in Probability and Statistics). Available from https://onlinelibrary.wiley.com/doi/book/10.1002/9780470567333

[12] van Dijk, C. E. et al. Type II diabetes patients in primary care: profiles of healthcare utilization obtained from observational data. *BMC Health Serv. Res.* **13**(1), 7 (2013). 23289605 10.1186/1472-6963-13-7 PMC3570342

[13] El Fakiri, F., Foets, M. & Rijken, M. Health care use by diabetic patients in the Netherlands: patterns and predicting factors. *Diabetes Res. Clin. Pract.* **61**(3), 199–209 (2003). 12965110 10.1016/s0168-8227(03)00116-5

[14] Seng, J. J. B. et al. Differential health care use, diabetes-related complications, and mortality among five unique classes of patients with type 2 diabetes in Singapore: A latent class analysis of 71,125 patients. *Diabetes Care* **43**(5), 1048–1056 (2020). 32188774 10.2337/dc19-2519 PMC7171941

[15] Garcia, T. B. et al. Agreement between self-reports and statutory health insurance claims data on healthcare utilization in patients with mental disorders. *BMC Health Serv. Res.* **23**(1), 1243 (2023). 37951906 10.1186/s12913-023-10175-6 PMC10640759

[16] Icks, A. et al. Agreement found between self-reported and health insurance data on physician visits comparing different recall lengths. *J. Clin. Epidemiol.* **82**, 167–172 (2017). 27825891 10.1016/j.jclinepi.2016.10.009

[17] Hoffmann, F. & Icks, A. Unterschiede in der Versichertenstruktur von Krankenkassen und deren Auswirkungen für die Versorgungsforschung: Ergebnisse des Bertelsmann-Gesundheitsmonitors. *Gesundheitswesen* **74**, 291–297 (2012). 21755492 10.1055/s-0031-1275711

[18] Hauner, H., Köster, I. & von Ferber, L. Prävalenz des Diabetes mellitus in Deutschland 1998–2001. Sekundärdatenanalyse einer Versichertenstichprobe der AOK Hessen/KV Hessen. *Dtsch. Arztebl.* **104**(41), A2799-2805 (2007). 10.1055/s-2003-812396 14673738

[19] Köster, I., Von Ferber, L., Ihle, P., Schubert, I. & Hauner, H. The cost burden of diabetes mellitus: The evidence from Germany—the CoDiM Study. *Diabetologia* **49**(7), 1498–1504 (2006). 16752168 10.1007/s00125-006-0277-5

[20] Linnenkamp, U. et al. Using statutory health insurance data to evaluate non-response in a cross-sectional study on depression among patients with diabetes in Germany. *Int. J. Epidemiol.* **49**(2), 629–637 (2020). 31990354 10.1093/ije/dyz278 PMC7266537

[21] Naldi, L. & Cazzaniga, S. Research techniques made simple: Latent class analysis. *J. Investig. Dermatol.* **140**(9), 1676-1680.e1 (2020). 32800180 10.1016/j.jid.2020.05.079

[22] Liebl, A. et al. Complications, co-morbidity, and blood glucose control in type 2 diabetes mellitus patients in Germany: Results from the CODE-2 ™ study. *Exp. Clin. Endocrinol. Diabetes.* **110**(01), 10–16 (2002). 11835119 10.1055/s-2002-19988

[23] von Ferber, L., Köster, I. & Hauner, H. Kosten der antihyperglykämischen Behandlung des Diabetes mellitus: Einfluss von Lebensalter, Therapieart und Komplikationsstatus: Ergebnisse der KoDiM-Studie 2001. *Med. Klin.* **101**(5), 384–393 (2006). 10.1007/s00063-006-1050-8 16685485

[24] Günster, C., Altenhofen, L. (eds) *Schwerpunkt: Chronische Erkrankungen*; [mit Online-Zugang]. Stuttgart: Schattauer, 354 (Versorgungs-Report, 2011).

[25] Thomas, G. Grobe, S. S., Joachim Szecsenyi. *Arztreport 2017 - Schriftenreihe zur Gesundheitsanalyse [Internet]*, Vol. 1. Asgard Verlagsservice GmbH; [cited 2020 Apr 23]. Available from https://www.barmer.de/blob/99196/40985c83a99926e5c12eecae0a50e0ee/data/dl-barmer-arztreport-2017.pdf.

[26] Liebl, A. et al. Kosten des Typ-2-diabetes in Deutschland. *DMW – Dtsch. Med. Wochenschr.* **126**(20), 585–589 (2001). 10.1055/s-2001-14102 11402924

[27] Sudore, R. L. et al. Limited literacy in older people and disparities in health and healthcare access: Limited literacy in older people and health disparities. *J. Am. Geriatr. Soc.* **54**(5), 770–776 (2006). 16696742 10.1111/j.1532-5415.2006.00691.x

[28] Paduch, A. et al. Psychosocial barriers to healthcare use among individuals with diabetes mellitus: A systematic review. *Prim. Care Diabetes.* **11**(6), 495–514 (2017). 28918199 10.1016/j.pcd.2017.07.009

[29] Baumeister, S. E. et al. Trends of barriers to eye care among adults with diagnosed diabetes in Germany, 1997–2012. *Nutr. Metab. Cardiovasc. Dis.* **25**(10), 906–915 (2015). 26298427 10.1016/j.numecd.2015.07.003

[30] Brown, A. F. Socioeconomic position and health among persons with diabetes mellitus: A conceptual framework and review of the literature. *Epidemiol. Rev.* **26**(1), 63–77 (2004). 15234948 10.1093/epirev/mxh002

[31] Rhodes, P., Nocon, A. & Wright, J. Access to diabetes services: The experiences of Bangladeshi people in Bradford, UK. *Ethinicty Health.* **8**(3), 171–188 (2003). 10.1080/1355785032000136407 14577994

[32] Kwan, J., Razzaq, A., Leiter, L. A., Lillie, D. & Hux, J. E. Low socioeconomic status and absence of supplemental health insurance as barriers to diabetes care access and utilization. *Can. J. Diabetes.* **32**(3), 174–181 (2008).

[33] Rothman, R. L. Influence of patient literacy on the effectiveness of a primary care-based diabetes disease management program. *JAMA* **292**(14), 1711 (2004). 15479936 10.1001/jama.292.14.1711

[34] Young, B. A. et al. Diabetes complications severity index and risk of mortality, hospitalization, and healthcare utilization. *Am. J. Manag. Care* **14**(1), 15–23 (2008). 18197741 PMC3810070

[35] Anderson, R. M. et al. Patient empowerment: Results of a randomized controlled trial. *Diabetes Care* **18**(7), 943–949 (1995). 7555554 10.2337/diacare.18.7.943

[36] Chang, H. Y., Weiner, J. P., Richards, T. M., Bleich, S. N. & Segal, J. B. Validating the adapted diabetes complications severity index in claims data. *Am. J. Manag. Care* **18**(11), 721–726 (2012). 23198714

[37] Ciechanowski, P. S., Katon, W. J. & Russo, J. E. Depression and diabetes: Impact of depressive symptoms on adherence, function, and costs. *Arch. Intern. Med.* **160**(21), 3278–3285 (2000). 11088090 10.1001/archinte.160.21.3278

[38] Ware, J., Kosinski, M. & Keller, S. D. A 12-item short-form health survey: Construction of scales and preliminary tests of reliability and validity. *Med. Care* **34**(3), 220–233 (1996). 8628042 10.1097/00005650-199603000-00003

[39] Weller, B. E., Bowen, N. K. & Faubert, S. J. Latent class analysis: A guide to best practice. *J. Black Psychol.* **46**(4), 287–311 (2020).

[40] Nylund, K. L., Asparouhov, T. & Muthén, B. O. Deciding on the number of classes in latent class analysis and growth mixture modeling: A Monte Carlo simulation study. *Struct. Equ. Model Multidiscip. J.* **14**(4), 535–569 (2007).

[41] Lanza, S. T. & Rhoades, B. L. Latent class analysis: An alternative perspective on subgroup analysis in prevention and treatment. *Prev. Sci. Off. J. Soc. Prev. Res.* **14**(2), 157–168 (2013). 10.1007/s11121-011-0201-1 PMC3173585 21318625

[42] Egede, L. E., Zheng, D. & Simpson, K. Comorbid depression is associated with increased health care use and expenditures in individuals with diabetes. *Diabetes Care* **25**(3), 464–470 (2002). 11874931 10.2337/diacare.25.3.464

[43] Vamos, E. P., Mucsi, I., Keszei, A., Kopp, M. S. & Novak, M. Comorbid depression is associated with increased healthcare utilization and lost productivity in persons with diabetes: A large nationally representative Hungarian population survey. *Psychosom Med.* **71**(5), 501–507 (2009). 19528291 10.1097/PSY.0b013e3181a5a7ad

[44] Yan, S. et al. Identifying heterogeneous health profiles of primary care utilizers and their differential healthcare utilization and mortality: A retrospective cohort study. *BMC Fam. Pract.* **20**(1), 54 (2019). 31014231 10.1186/s12875-019-0939-2 PMC6477732

[45] Baumert, J., Paprott, R., Du, Y., Heidemann, C. & Scheidt-Nave, C. Selbsteingeschätzte Versorgungsqualität bei Erwachsenen mit diagnostiziertem Diabetes in Deutschland [Internet]. Robert Koch-Institut; 2021 [cited 2024 Oct 29]. Available from https://edoc.rki.de/handle/176904/8395

[46] Safieddine, B., Sperlich, S., Beller, J., Lange, K. & Geyer, S. Socioeconomic inequalities in type 2 diabetes comorbidities in different population subgroups: trend analyses using German health insurance data. *Sci. Rep.* **13**(1), 10855 (2023). 37407649 10.1038/s41598-023-37951-y PMC10322827

[47] IGES Institut GmbH. Bewertung der Kodierqualität von vertragsärztlichen Diagnosen [Internet]. Available from https://www.gkv-spitzenverband.de/media/dokumente/krankenversicherung_1/aerztliche_versorgung/verguetung_und_leistungen/klassifikationsverfahren/9_Endbericht_Kodierqualitaet_Hauptstudie_2012_12-19.pdf

