# Supplementary material for: Linked survey and statutory health insurance data evaluating healthcare utilization patterns and associated factors of persons with diabetes in Germany – latent class analysis
Source: Sci Rep. 2025 Apr 4;15:11646. doi: 10.1038/s41598-025-95514-9 (PMC11971297; doi:10.1038/s41598-025-95514-9)
Supplement: Supplementary file 1 — Supplementary Material 1 [file 41598_2025_95514_MOESM1_ESM.docx]

Appendix for Manuscript titled

“Linked survey and statutory health insurance data evaluating healthcare utilization patterns and associated factors of persons with diabetes in Germany – latent class analysis”

| Appendix Table 1 Statistical parameters for models with different numbers of classes per variant of LCA without covariates | | | | | | |
| --- | --- | --- | --- | --- | --- | --- |
| Number of classes | AIC | BIC | CAIC | aBIC | Entropy | p-value |
| 1 | 519.3 | 562.6 | 570.6 | 537.2 | 1 | 0 |
| 2 | 250.3 | 342.4 | 359.4 | 288.4 | 0.4 | 0.02 |
| 3 | 250.4 | 391.2 | 417.2 | 308.6 | 0.6 | 0.04 |
| 4 | 254.3 | 443.8 | 478.8 | 332.6 | 0.7 | 0.06 |
| Information criteria (smaller values indicate better fitting): AIC = Akaike information criterion, BIC = Bayesian information criterion, CAIC = Consistent Akaike information criterion, aBIC = Sample size adjusted BIC,  Entropy = relative entropy (range of values 0-1, higher values of entropy indicate better classification of individuals)  p-values were determined based on likelihood ratio chi-square tests | | | | | | |

| Appendix *Table 2. Characteristics of participants assigned to the different classes* | | | | |
| --- | --- | --- | --- | --- |
|  | Class 1 –  Low users | Class 2 –  Low users with ophthalmologist visits | Class 3 –  High users | Class 4 –  High users with mental healthcare |
| Characteristics n (%) / (M ± SD) |  |  |  |  |
| **Sample size (%)*** | 289 (21.7) | 585 (43.9) | 342 (25.7) | 116 (8.7) |
| Age (years) | 62.3 ± 11.2 | 67.7 ± 9.2 | 70.4 ± 7.5 | 61.7 ± 11.9 |
| Sex (female) | 87 (30.1) | 222 (38.0) | 121 (35.4) | 61 (52.6) |
| Years of education |  |  |  |  |
| *≥14 years* | 84 (29.1) | 131 (22.4) | 66 (19.3) | 17 (14.7) |
| *11-13 years* | 165 (57.1) | 348 (59.5) | 188 (55.0) | 66 (56.9) |
| *≤10 years* | 40 (13.8) | 106 (18.1) | 88 (25.7) | 33 (28.4) |
| Country of birth (Germany) | 257 (88.9) | 524 (89.6) | 309 (90.4) | 95 (81.9) |
| Partner (yes) | 230 (79.6) | 476 (81.4) | 273 (79.8) | 89 (76.7) |
| Employment (yes) | 122 (42.2) | 146 (25.0) | 54 (15.8) | 36 (31.0) |
| Type of diabetes (type 2) | 249 (86.2) | 538 (92.0) | 328 (95.9) | 96 (82.8) |
| Diabetes duration (≥10 years) | 111 (38.4) | 302 (51.6) | 208 (60.8) | 49 (42.2) |
| aDCSI | 1.4 ± 1.4 | 2.1 ± 1.6 | 4.2 ± 2.2 | 2.6 ± 1.9 |
| DMP Member (yes) | 210 (72.7) | 505 (86.3) | 310 (90.6) | 96 (82.8) |
| Diabetes training (yes) | 28 (9.7) | 69 (11.8) | 69 (20.2) | 23 (19.8) |
| PCS12 | 45.4 ± 9.2 | 44.4 ± 10.2 | 36.5 ± 10.7 | 35.6 ± 10.6 |
| MCS12 | 51.6 ± 9.8 | 52.5 ± 8.6 | 49.4 ± 10.6 | 38.0 ± 11.9 |
| Depression (yes) | 30 (10.4) | 48 (8.2) | 71 (20.8) | 85 (73.3) |
| * Participants were assigned to the latent class with the highest probability of individual class membership, thus it differs to the class prevalence reported in Figure 1. M = Mean, SD = standard deviations, aDCSI = adjusted diabetes severity index, DMP = disease management program PCS12 = Physical Component Summary score of the Short Form-12 Health Survey, MCS12 = Mental Component Summary score of the Short Form-12 Health Survey | | | | |

| Appendix *Table 3. Probability of health care utilization of study participants assigned to the different classes* | | | | |
| --- | --- | --- | --- | --- |
|  | Class 1 –  Low users | Class 2 –  Low users with ophthalmologist visits | Class 3 –  High users | Class 4 –  High users with mental healthcare |
| **Healthcare utilization** (±SD) |  |  |  |  |
| GP visits (>14) | 0.19 (± 0.03) | 0.30 (± 0.03) | 0.80 (± 0.03) | 0.53 (± 0.07) |
| Emergncy care (≥1) | 0.09 (± 0.02) | 0.07 (± 0.01) | 0.22 (± 0.03) | 0.22 (± 0.05) |
| Hospitalization (≥1) | 0.14 (± 0.03) | 0.12 (± 0.02) | 0.63 (± 0.04) | 0.51 (± 0.07) |
| Cardiologist visits (≥1) | 0.12 (± 0.02) | 0.13 (± 0.02) | 0.44 (± 0.03) | 0.27 (± 0.05) |
| Nephro-/Neurologist visit (≥1) | 0.06 (± 0.02) | 0.05 (± 0.01) | 0.24 (± 0.03) | 0.11 (± 0.04) |
| Ophthalmologist visits |  |  |  |  |
| *0* | 0.93 (± 0.09) | 0.03 (± 0.04) | 0.23 (± 0.03) | 0.26 (± 0.06) |
| *1* | 0.06 (± 0.09) | 0.55 (± 0.05) | 0.30 (± 0.03) | 0.45 (± 0.06) |
| ≥2 | 0.00 (± 0.01) | 0.43 (± 0.03) | 0.47 (± 0.03) | 0.29 (± 0.06) |
| Mental health sector visits (≥1) | 0.06 (± 0.02) | 0.04 (± 0.01) | 0.09 (± 0.03) | 0.99 (± 0.02) |
| SD=standard deviations, GP=General Practitioner | | | | |
